# Case Study: Misdiagnosis of Nonhemolytic *Staphylococcus aureus* Isolates from Cases of Bovine Mastitis as Coagulase-Negative Staphylococci

**DOI:** 10.3390/ani11020252

**Published:** 2021-01-20

**Authors:** Valerie E. Ryman, Felicia M. Kautz, Steve C. Nickerson

**Affiliations:** Department of Animal and Dairy Science, University of Georgia, Athens, GA 30605, USA; fkautz2@gmail.com (F.M.K.); scn@uga.edu (S.C.N.)

**Keywords:** misdiagnosis, *Staphylococcus aureus*, CNS, NAS, hemolysis

## Abstract

**Simple Summary:**

Correct identification of mastitis-causing pathogens is key in lactating dairy cattle as management decisions depend on correct diagnoses. One of the most concerning pathogens is *Staphylococcus aureus*. In simple laboratory set-ups, presumptive identification is performed by assessing the bacterial colonies’ physical characteristics. In the absence of notable characteristics, isolates are deemed to be non-aureus isolates. Over a decade, however, it became evident that *S. aureus* identifications may be incorrect as further testing was performed on milk and mammary secretion samples. This short report details the laboratory findings from isolates collected on a Georgia dairy farm over a 10-year period. Almost 64% of isolates that were deemed to be non-aureus isolates were actually identified as *S. aureus*. Just over 26% were correctly identified. This communication is a cautionary tale to laboratories, but especially dairy producers performing simple on-farm microbiological procedures. Further testing or more advanced testing may need to be performed to develop appropriate control, prevention, and treatment plans.

**Abstract:**

*Staphylococcus aureus* is one of the most concerning mastitis-causing pathogens in dairy cattle. Using basic microbiological techniques, *S. aureus* is typically identified by colony characteristics and hemolysis on blood agar where isolates without hemolysis are typically considered to be coagulase-negative staphylococci (CNS) isolates. Herein, we present a decade-long case study where suspected *S. aureus* isolates from one Georgia dairy farm were further tested to confirm presumptive identification. Presumptive identification of bacterial growth from 222 mammary secretions from bred Holstein heifers and lactating cows was conducted at the time of collection. Presumptive identification of *S. aureus* on blood agar was based on observation of colony morphology, color, and presence or absence of a broad zone of incomplete hemolysis and a smaller zone of complete hemolysis at 48 h. Those without hemolysis were presumptively characterized as CNS. All isolates were further plated on mannitol salt agar and a coagulase test was performed. A positive for both of these tests together was deemed to be *S. aureus*. A selection of isolates was tested using API^®^ Staph to biochemically confirm *S. aureus* identification. Data showed that 63.96% of isolates presumed to be CNS isolates were identified as *S. aureus*, 9.46% of isolates presumed to be CNS isolates were identified as coagulase-positive staphylococci (CPS) species (but not *S. aureus*), and 26.58% of samples that were presumed to be CNS isolates were identified correctly.

## 1. Introduction

*Staphylococcus aureus* continues to be a major cause of bovine mastitis. Managing this form of mastitis via preventative measures has been shown to be economically advantageous [1,2]. In addition, treatment of *S. aureus* mastitis during lactation is economically justified in many situations [3]. If therapy is delayed, the duration of infection increases and is accompanied by extended periods of bacterial shedding, elevated somatic cell counts (SCCs), scar tissue formation, and spread of infection to multiple quarters within a cow, all of which are associated with low cure rates [4,5] and transmission to other cows in the herd [6].

Presumptive identification of *S. aureus* on blood agar is largely based on observation of colony morphology, color, and hemolysis, typically both complete and incomplete. Because fewer than 5% of isolates express no hemolysis, it is widely accepted that the presence of complete and/or incomplete provides a strong diagnosis of *S. aureus*, where lack of hemolysis indicates a non-aureus staphylococci (NAS). The NAS are typically distinguished from *S. aureus* as being coagulase-negative staphylococci (CNS). A coagulase test, however, will not differentiate *S. aureus* from the coagulase-positive species *Staphylococcus intermedius* or the coagulase-variable species *Staphylococcus hyicus* in the absence of hemolysis. Thus, an additional step commonly taken is to plate isolates on mannitol salt agar (MSA) to determine the capability of the isolate to ferment mannitol (*S. aureus* is capable, whereas most NAS, including CNS, are not). Utilizing the MSA test in combination with the tube coagulase test enhances the specificity and sensitivity of identification [7].

In past work assessing mastitis prevalence in Georgia dairies, it was noted that isolates presumptively identified as CNS were associated with the high SCCs typically observed with *S. aureus*, e.g., >500,000 cells/mL. Because of the elevated SCCs, further testing was carried out, which revealed these isolates were coagulase positive as well as MSA positive, indicating presumptive identification as *S. aureus*, rather than CNS, which prompted continued collection and testing of isolates. The purpose of this investigation was to determine the prevalence of these non-hemolytic *S. aureus* isolates that were misclassified as CNS. The correct identification is important from a mastitis management standpoint, as all cows diagnosed with *S. aureus* mastitis should be managed much differently than those with CNS mastitis, such as separating from the lactating herd to prevent contagious spread of *S. aureus*.

## 2. Materials and Methods

A total of 222 mammary secretions had been previously aseptically collected from bred Holstein heifers and lactating cows. Bred Holstein heifers and lactating cows were housed and cared for in the University of Georgia Teaching Dairy [8]. The average herd size across the 10 years of data collection was approximately 111 lactating cows housed in a conventional freestall barn with an average milk yield of 66.1 lbs/cow and an average somatic cell count (SCC) of 273,500 cells/mL. Lactating dairy animals were milked 2 times/day in a double-6 herringbone parlor. After collection, mammary secretions were plated on trypticase soy agar plates with 5% sheep blood using an aseptic technique. Plates were incubated for 48 h at 37 °C and then visually inspected for the presence of colony growth. Through presumptive identification based on colony color, morphology, and presence of hemolysis (discussed in the succeeding paragraph), a determination was made regarding whether isolates were *S. aureus* or other staphylococci (such as CNS).

Colonies that were creamy to grayish-white or yellow, smooth, circular, catalase positive, and that exhibited a narrow zone of complete hemolysis and a wide zone of incomplete hemolysis, were identified as *S. aureus*. Colonies identified as CNS because no hemolysis was present were further tested with the tube coagulase test and the MSA test. The tube coagulase test was performed according to basic microbiological procedures. To perform the MSA test, an MSA plate streaked with bacteria was incubated for 24 h at 37 °C. The MSA test was deemed positive if the bacteria colonized the agar surface and the plate medium turned bright yellow, indicating that the pathogen was able to grow in a high saline environment and fermented mannitol to produce the color change. If the bacteria colonized the agar surface, but the medium did not change color (remained pink), the test was deemed negative (for mannitol fermentation).

A coagulase-positive, MSA-positive result was categorized as *S. aureus* given its growth requirements and characteristics, despite the lack of hemolysis. A coagulase-negative, MSA-negative isolate was termed a CNS. A coagulase-positive, MSA-negative isolate was termed a coagulase-positive staphylococci (CPS). We also tested a subset of the nonhemolytic *S. aureus* isolates (approximately 60%) using the API^®^Staph system (bioMerieux, Inc., Marcy l’Etoile, France) according to manufacturer’s specifications to further support our *S. aureus* identification. All isolates tested were determined to be *S. aureus*. The API^®^Staph system was also used to test a selection of the CNS and CPS isolates to determine various species of staphylococci detected from cases of mastitis. 

The distribution of infections amongst the quarters was described and analyzed by the non-parametric Kruskal–Wallis test with Dunn’s post-hoc testing.

## 3. Results and Discussion

**Figure 1:**
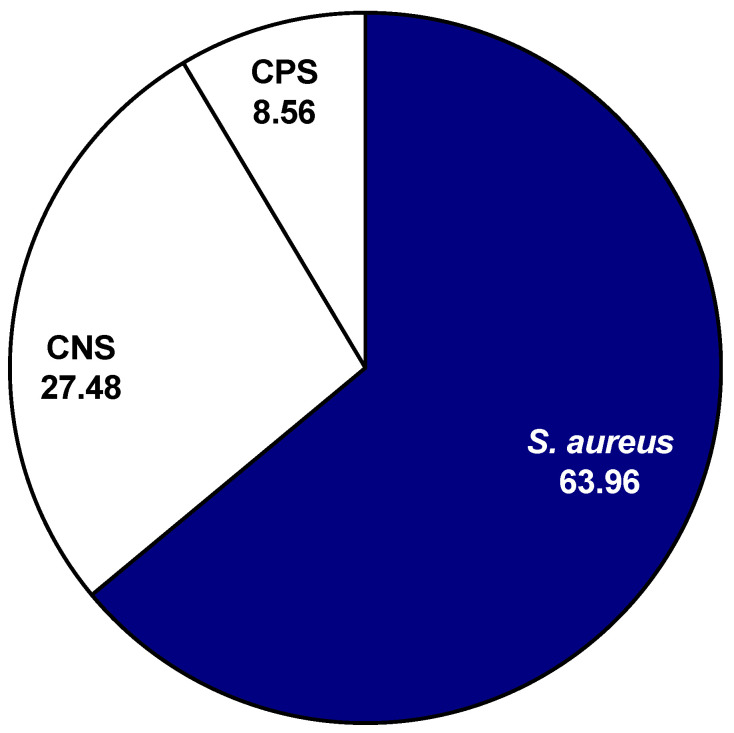
Identification of isolates (%) presumed to be coagulase-negative staphylococci (CNS) species.

**Figure 2:**
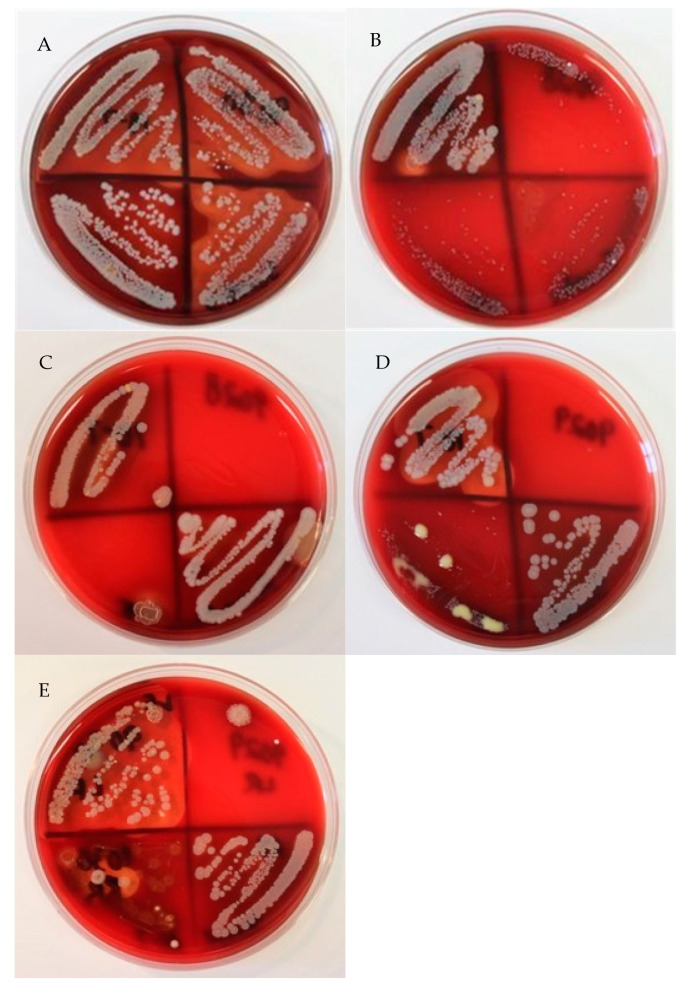
(**A**) Four quarter mammary secretions showing 3 quarters with hemolytic *S. aureus* (top left, top right, and bottom right) and 1 quarter nonhemolytic *S. aureus* (bottom left). (**B**) Four quarter mammary secretions showing 1 quarter with nonhemolytic *S. aureus* (top left). There is 1 area of hemolytic contamination (small Gram-positive *Bacillus*) in this mammary secretion streak. Though outside the scope of the report, the remaining 3 quarters show environmental streptococci. (**C**) Four quarter mammary secretions showing 1 quarter nonhemolytic *S. aureus* (top left) and 1 quarter coagulase-positive staphylococci (CPS) (*S. intermedius*, bottom right). (**D**) Four quarter mammary secretions showing 1 quarter hemolytic *S. aureus* (top left) and 1 quarter nonhemolytic *S. aureus* (bottom right). These particular nonhemolytic *S. aureus* have distinct cream-colored centers on a greyish-white colony. (**E**) Four quarter mammary secretions showing 1 quarter hemolytic *S. aureus* (yellower in color than other hemolytic *S. aureus* found, top left) and 1 quarter nonhemolytic *S. aureus* (bottom right). These particular nonhemolytic *S. aureus* also have distinct cream-colored centers on a greyish-white colony (similar to those seen in Figure 2D).

After plating and testing 222 mammary secretions, the data showed that 63.96% (142 out of 222) of the isolates presumed to be CNS isolates were identified as *S. aureus* (Figure 1). Additionally, 8.56% (19 out of 222) of the isolates presumed to be CNS isolates were identified as CPS species. A total of 27.48%, (61 out 222) of the samples that were presumed to be CNS isolates were identified correctly. In the current study, not all nonhemolytic *S. aureus* colonies were consistent in appearance, but many of them were grayish-white without areas of hemolysis surrounding the colonies (Figure 2). The agar displayed a green hue (alpha hemolysis) behind and around the colonies. The centers of many atypical *S. aureus* isolates were raised and exhibited a slightly different color than the rest of the colony. 

According to the Laboratory Handbook on Bovine Mastitis [9], *S. aureus* isolates are presumptively identified as follows: Isolates are Gram-positive as well as catalase-positive cocci. Culturing of these isolates reveals creamy, grayish-white to golden-yellow colonies with distinct areas of incomplete hemolysis, with or without areas of narrow complete hemolysis. However, as reported in this study, the *S. aureus* isolates identified were not hemolytic, resulting in incorrect presumptive identification as CNS isolates. Although it is noted in the Laboratory Handbook on Bovine Mastitis that not all isolates will demonstrate hemolysis, the presence of hemolysis for *S. aureus* identification is still a commonly held belief, especially in on-farm milk culturing.

**Figure 3:**
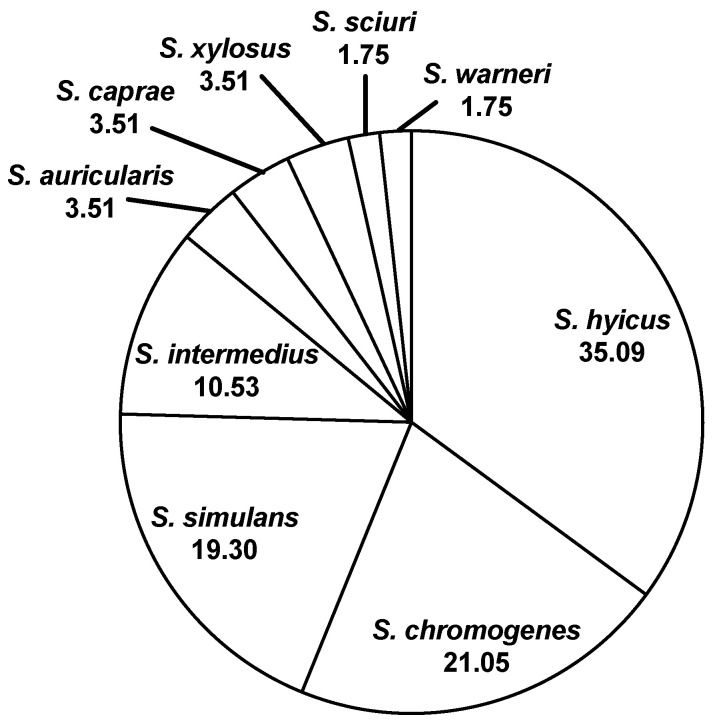
Identification (%) of non-aureus isolates (CNS and CPS).

Typical CNS isolates are nonpigmented, creamy grayish-white, tan, or golden-yellow colonies. These isolates do not display complete hemolysis typical of *S. aureus*; however, some species of CNS have been reported to display diffuse zones of hemolysis. Thus, isolates lacking the typical hemolytic patterns are presumptively identified as CNS species. As stated in the Materials and Methods, a selection of isolates was tested utilizing the API^®^Staph system. The genus and species identification of those that were not *S. aureus* are shown in Figure 3. An incorrect diagnosis could directly impact managerial decisions for cow management. Depending on the mastitis prevention and control plan for an operation, cows diagnosed with *S. aureus* may be considered for extended intramammary antibiotic therapy, alternative intramammary antibiotics, separation from herd mates, and altered milking order to prevent spread, reevaluation of fly control methods, and culling [10].

**Figure 4:**
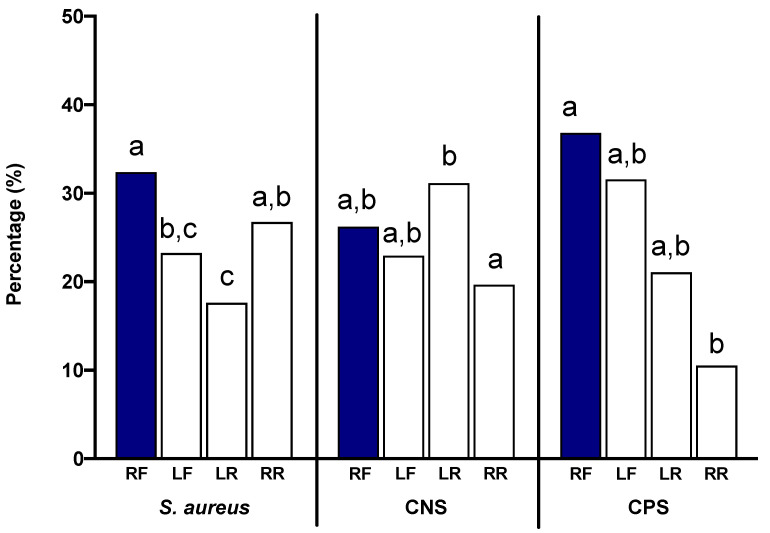
Distribution of non-hemolytic *S. aureus*, CNS, and CPS infection among quarters. Letters above bars represent differences within each pathogen group. Letters that are similar are not different (*p* > 0.05), whereas letters that differ are significantly different (*p* < 0.05).

Given the concerns surrounding *S. aureus* and its impact on mammary health and milk production, we were also interested in determining whether a relationship existed between these nonhemolytic *S. aureus* isolates and afflicted quarters of the dairy animal. Previous research found a relationship between *S. aureus* infection and the quarter affected, where front teats were more likely to be infected with *S. aureus* compared to rear teats [11]. Therefore, we investigated this potential dynamic in the current study. In quarters infected with nonhemolytic *S. aureus*, 32.39% of the isolates were collected from the right front (RF) quarters, 23.24% from the left front (LF) quarters, 17.61% from the left rear (LR) quarters, and 26.76% from the right rear (RR) quarters (Figure 4). The left quarters displayed fewer nonhemolytic infections numerically; however, only the LR statistically differed from the RF and RR, with fewer identified *S. aureus* infections (*p* < 0.05). In quarters infected with CNS, 26.23% of the isolates were collected from the RF quarters, 22.95% from the LF quarters, 31.15% from the LR quarters, and 19.67% from the RR quarters. In contrast to the *S. aureus* infections, the LR CNS cases were significantly greater than the RR ones (*p* < 0.05), but not different from the RF or LF ones. Lastly, in quarters infected with CPS, 36.84% of the isolates were collected from the RF quarters, 31.58% from the LF quarters, 21.05% from the LR quarters, and 10.53% from the RR quarters. The RF CPS cases were significantly greater than the RR cases.

We expected to see a greater percentage of *S. aureus* infections in the front quarters, as was observed in previous research. The front quarters/teats are in close proximity to the umbilical region of the animal, which is where fly populations tend to be greatest. Horn flies have been identified as important vectors in the spread of *S. aureus*, especially in heifers [12]. Though the RF quarters did represent a greater percentage compared to the LR, the RF was also greater than the LF, a result that is not fully understood. Additional isolates of CPS species would be needed to fully evaluate the quarter-level relationships identified in the current study, especially given the significant difference between the LR and RR. There is not a clear biological reason why prevalence of infection would be different in the left side versus the right side of the dairy animal. Behavioral investigations (animal and human) could reveal animals that prefer to lay on one side, and thus put one side of their mammary gland at risk over the other, or perhaps these animals prefer to enter the milking parlor on the same side each time, which happens to be the farthest away from the milker, and that far side is less thoroughly cleaned as is anecdotally observed during farm visits and milker trainings. However, this is conflicting when you compare the rear quarters of *S. aureus* with the rear quarters of CNS. Why does this happen? Ultimately, it is unclear why these differential associations exist between quarters and pathogens; nonetheless, the results are important to report. Interpretation of the results is limited given the combination of heifer secretions and milk samples, as well as the combination of subclinical and clinical cases; thus, the quarter-level differences require further investigation.

An important note for the current study is that the isolates were collected from one Georgia farm. It is possible, and quite likely, that the extremely high rate of nonhemolytic *S. aureus* isolates was specific to this farm and the circulating strains on that unit. However, many dairy operations that do not actively culture and/or are just new to implementing a culturing program, especially on-farm, may not know the microbiological make-up of their mastitis-causing pathogen profile. This report serves as a word of caution, and perhaps suggestion that further simple microbiological tests are performed to increase confidence in diagnosis. On-farm milk culturing is a low-cost, valuable tool in mastitis control plans; thus, future studies should focus on performing collections and microbiological analysis on various farms throughout the region to determine the potential depth and breadth of this issue.

When possible, consideration should be taken to conduct identification using gold standard techniques that surpass basic microbiological techniques. For example, DNA and protein technology enable rapid, accurate diagnosis of common mastitis-causing pathogens, such as *S. aureus* and many CNS/CPS [13]. Moreover, these technologies allow for differentiation between CNS and CPS species as well. As we learn more about the immune responses of bacteria and their response to various treatment plans, it may become important to not only differentiate between *S. aureus* and CNS/CPS, but also *S. hyicus* vs. *S. chromogenes*, as an example. This is already the case for environmental streptococci such as *Streptococcus uberis* and *S. dysgalactiae*. More than 5 years ago, DNA-based techniques rose in popularity and are still used in many labs today. DNA-based technology provides improved accuracy over microbiological culturing and biochemical assays in a shorter period of time. Several companies currently market on-farm PCR systems to identify a few of the most predominant mastitis pathogens, though implementation of these on-farm systems are cost-prohibitive for many operations. While DNA-based technologies are superior to culturing and simple biochemical assays, there are still limitations in that only the most common bacteria are capable of being identified (currently) and both live and dead bacteria are detected, which could complicate real-time antibiotic therapy strategies [13]. In light of these limitations, matrix-assisted laser desorption/ionization time-of-flight mass spectrometry (MALDI-TOF MS) has become the preferable choice for rapid, accurate diagnosis because of the comparative databases for identification [14]. While there are still considerations for the challenges of MALDI-TOF (i.e., the sample must be a pure culture, free from contaminating pathogens), this limitation does not preclude its value moving forward in identification of mastitis-causing bacteria, as subcultures of causative pathogens following visual assessment can be garnered. 

## 4. Conclusions

Given the results of this study, the current guidelines for presumptive identification of *S. aureus* and CNS are inadequate. The completion of coagulase tests and MSA tests eliminates such speculation and should be conducted to differentiate CNS from *S. aureus*. The correct identification is important from a mastitis management standpoint, as all cows with *S. aureus* mastitis and with elevated SCCs should be handled properly and in a timely manner to prevent the spread of this contagious pathogen. Ultimately, dairy producers should consider submitting samples to laboratories that can verify identification using either DNA-based technology or proteomics, such as PCR or MALDI-TOF.

## Data Availability

The data reported in this study is available upon request from the corresponding author.
